# STAT3 at the tumor-immune interface: mechanisms of immune escape and therapeutic opportunities

**DOI:** 10.3389/fimmu.2026.1850359

**Published:** 2026-05-19

**Authors:** Xuanya Cheng, Haoren Jing, Mingqing Zhang

**Affiliations:** 1 Tianjin University of Traditional Chinese Medicine, College of Traditional Chinese Medicine, Tianjin, China; 2 Department of Colorectal Surgery, Tianjin Union Medical Center, The First Affiliated Hospital of Nankai University, Tianjin, China; 3 Tianjin Institute of Coloproctology, Tianjin Union Medical Center, The First Affiliated Hospital of Nankai University, Nankai University, Tianjin, China

**Keywords:** immune escape, immunotherapy, metabolic reprogramming, PD-L1, stat3, targeted therapy, tumor microenvironment, tumor-immune interface

## Abstract

Immune escape remains a major barrier to durable cancer immunotherapy. Although checkpoint blockade has transformed cancer treatment, resistance commonly reflects broader tumor-intrinsic and microenvironmental programs that sustain immune dysfunction. At this interface, STAT3 emerges as a central organizing node. Beyond its canonical role in inflammatory and oncogenic signaling, STAT3 links tumor cell plasticity, immune suppression, and metabolic adaptation across the tumor ecosystem. In tumor cells, STAT3 promotes stemness, survival, checkpoint ligand expression, impaired antigen presentation, and immunosuppressive secretomes. In immune compartments, it drives regulatory T cell expansion, myeloid-derived suppressor cell accumulation, tumor-associated macrophage polarization, and dendritic cell dysfunction, thereby stabilizing an immune-resistant niche. STAT3 also reinforces immune escape through metabolic rewiring and multicellular feed-forward circuits. These features make STAT3 an attractive but challenging therapeutic target. Here, we discuss how STAT3 functions at the tumor-immune interface to coordinate immune escape and highlight therapeutic opportunities for targeting this axis in cancer.

## 1 Introduction

Tumor immunity refers to the capacity of the host immune system to recognize and eliminate malignant cells, whereas the tumor immune microenvironment critically shapes the magnitude and quality of antitumor responses. Together, these processes influence tumor initiation, progression, immune escape, and therapeutic response (1). Under physiological conditions, immune surveillance can constrain malignant transformation; however, during tumor evolution, cancer cells acquire the ability to evade immune recognition, attenuate immune attack, and induce immune tolerance by rewiring both tumor-intrinsic programs and the surrounding microenvironment. Immune escape has therefore emerged as a central mechanism underlying disease progression, therapeutic failure, and limited long-term benefit from cancer immunotherapy (1).

**Figure 1:**
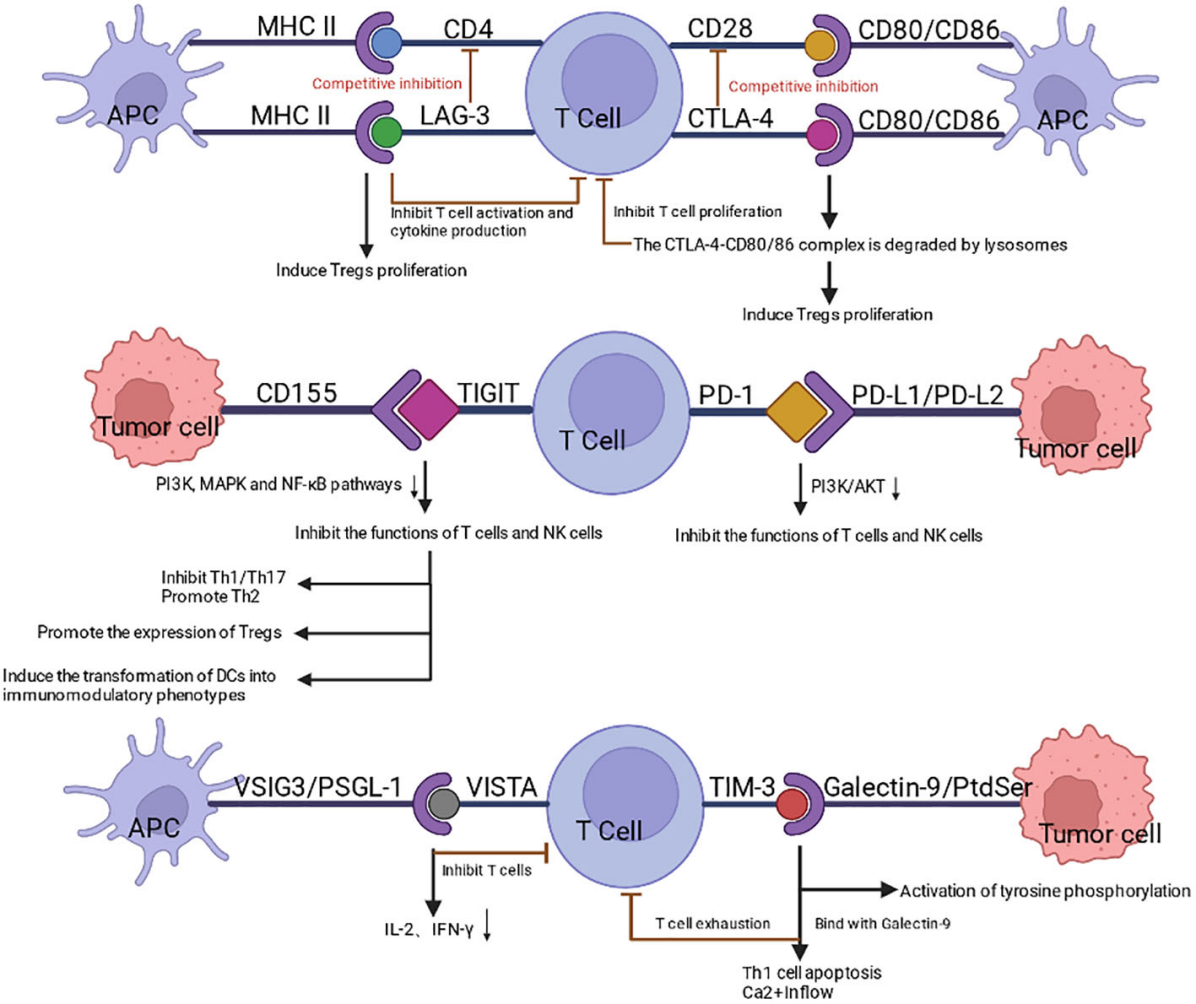
Major inhibitory receptor-ligand axes involved in tumor immune evasion and current immunotherapy. This schematic summarizes six representative inhibitory receptor-ligand axes that regulate T cell function and contribute to tumor immune escape. In the upper module, peptide-MHC II complexes on antigen-presenting cells (APCs) are recognized by the T cell receptor (TCR) on CD4^+^ T cells, with CD4 serving as a co-receptor to support T cell activation, whereas LAG-3 competitively suppresses this process and promotes Treg expansion. CD80/CD86 similarly interacts with CD28 to provide co-stimulation but can also bind CTLA-4, thereby restraining T cell proliferation and favoring Treg-mediated suppression. In the middle module, CD155 on tumor cells binds TIGIT on T cells, dampening T/NK cell activity, reshaping T helper cell responses, and promoting the expansion of Tregs and immunoregulatory DCs. PD-L1/PD-L2 on tumor cells likewise engages PD-1 on T cells to suppress T/NK cell function. In the lower module, VISTA mediates inhibitory signaling through context-dependent ligand interactions, including reported interactions with VSIG3 and PSGL-1, thereby suppressing T cell activation and cytokine production, whereas TIM-3 integrates signals from multiple ligands, including Galectin-9, phosphatidylserine, CEACAM1, and HMGB1, contributing to T cell exhaustion, impaired effector function, and immunosuppressive remodeling of the tumor microenvironment. Collectively, these checkpoint pathways establish an immunosuppressive microenvironment that enables tumor immune escape. Created in BioRender. Cheng, X. (2026) https://BioRender.com/ytfqqke.

The clinical success of immune checkpoint blockade has transformed cancer therapy and demonstrated that reactivating antitumor immunity can produce durable benefit in a subset of patients (2). Nevertheless, immunotherapy remains limited by primary and acquired resistance, incomplete response durability, and immune-related toxicities (3). Recent cellular immunotherapy studies likewise show that meaningful clinical responses may be accompanied by prolonged immune dysregulation, infections, autoimmune manifestations, and on-target/off-tumor toxicity when normal immune compartments are persistently perturbed (4, 5). These limitations indicate that immune escape is not driven by a single inhibitory pathway but rather by coordinated signaling networks that integrate tumor cell plasticity, stromal remodeling, and immune dysfunction. Identifying such nodal regulators is therefore essential for improving therapeutic precision and expanding the benefit of immunotherapy (3). Because checkpoint-mediated suppression remains a dominant mechanism of tumor immune evasion, **Figure 1** summarizes the major inhibitory receptor–ligand axes underpinning current immunotherapy and their immunosuppressive effects. Yet checkpoint signaling alone does not fully explain how immune-resistant tumor ecosystems are established and maintained. Among the candidate nodal regulators that connect these processes, STAT3 is particularly compelling.

STAT3 is activated downstream of cytokines, growth factors, and inflammatory mediators and coordinates programs governing proliferation, survival, differentiation, immunity, and inflammation. Once activated, Janus kinases (JAKs) phosphorylate STAT3, triggering its dimerization, nuclear translocation, and transcriptional control of target genes. Although transient STAT3 activation contributes to normal tissue homeostasis and immune regulation, persistent STAT3 signaling is a recurrent feature of tumors and inflammatory disorders and has therefore emerged as an attractive therapeutic target (6, 7). Importantly, STAT3 is not merely another oncogenic signaling pathway. Rather, it acts as a context-dependent signaling hub at the tumor-immune interface. In tumor cells, STAT3 supports survival, stemness, metabolic adaptation, and resistance to immune-mediated killing. In immune and stromal compartments, it promotes tolerogenic antigen presentation, T cell dysfunction, myeloid immunosuppression, and cytokine networks that stabilize an immune-resistant niche (6, 7). This dual activity places STAT3 in a unique position to link tumor-intrinsic plasticity with microenvironmental rewiring.

In this Review, we argue that STAT3 functions as a central organizer of cancer immune escape. We first summarize how STAT3 is activated and integrated with other signaling networks, then examine how it drives immune evasion through tumor-cell-intrinsic and immune-cell-intrinsic mechanisms. We next discuss how STAT3-dependent metabolic reprogramming and multicellular feedback circuits reinforce immune resistance, and finally consider how these insights can be exploited therapeutically through biomarker-guided and mechanism-based intervention strategies.

## 2 STAT3 signaling at the tumor-immune interface

To understand how STAT3 functions as an organizer of immune escape, it is first necessary to define the signaling modes through which it is activated within the tumor microenvironment. Interleukin-6 (IL-6) is a pleiotropic cytokine and a principal upstream driver of STAT3 signaling. Produced by tumor, stromal, and immune cells, IL-6 regulates not only hematopoietic and immune functions but also tumor proliferation, survival, invasion, and metastasis (8). In the canonical pathway, IL-6 binds to membrane-bound IL-6 receptor α (IL-6Rα, gp80) and the signal-transducing receptor gp130, forming a receptor complex that activates JAK2. Activated JAK2 then autophosphorylates and generates phosphotyrosine docking sites that recruit STAT3. STAT3 is subsequently phosphorylated, dimerizes, translocates into the nucleus, and binds STAT3-responsive elements in target genes, thereby controlling proliferation, differentiation, apoptosis, and immune regulation (8, 9). In many tumors, sustained activation of this pathway induces IL-6 itself, establishing a feed-forward circuit that continuously amplifies oncogenic and immunosuppressive signaling.

**Figure 2:**
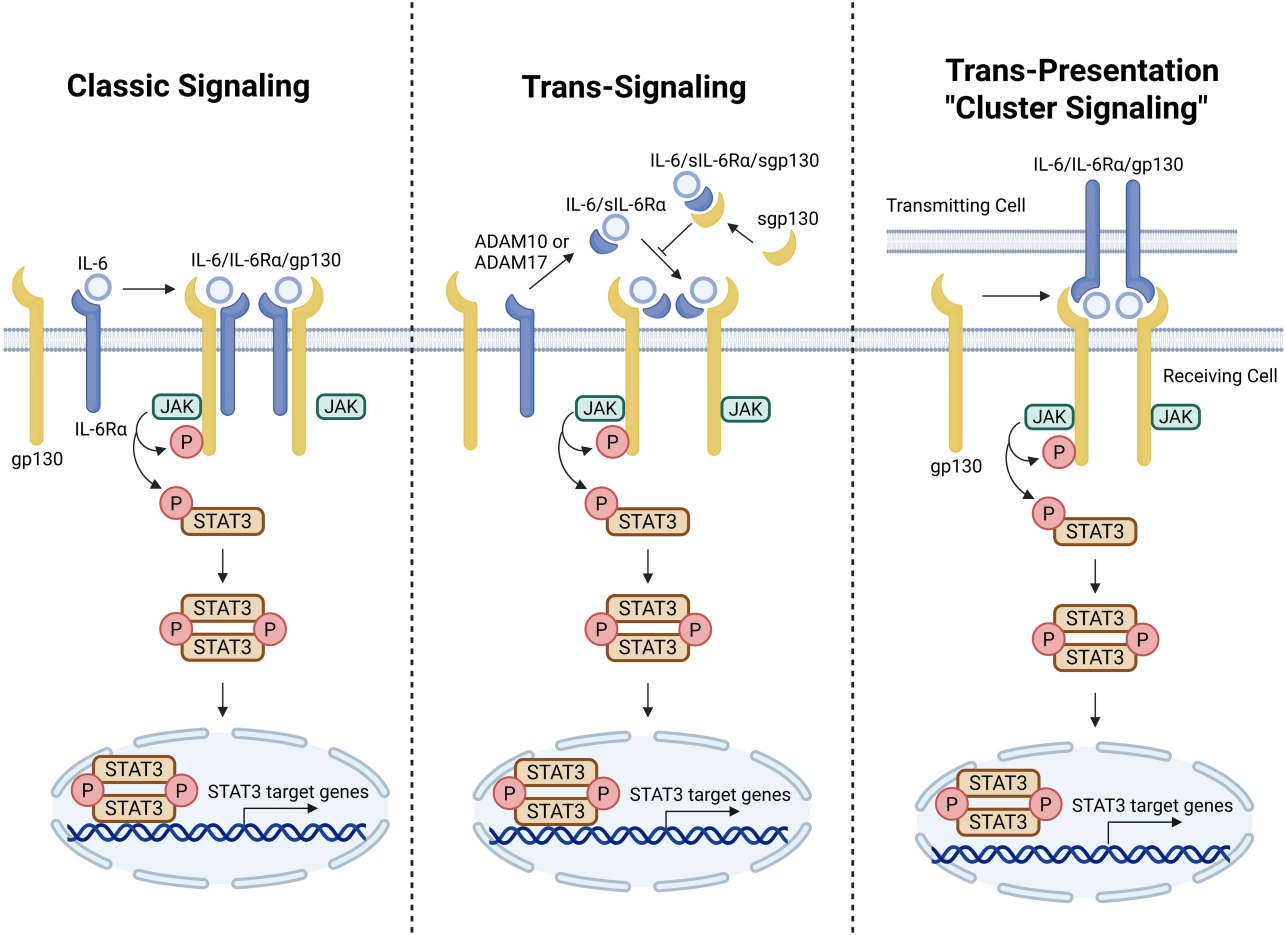
Canonical and non-canonical activation modes of the IL-6/JAK/STAT3 pathway. In canonical signaling, IL-6 binds membrane-bound IL-6Rα and gp130 to form a signaling complex that activates JAKs, phosphorylates STAT3, and drives its nuclear translocation and transcriptional activity. In trans-signaling, IL-6 first complexes with soluble IL-6Rα and then stimulates gp130-expressing cells that lack membrane-bound IL-6Rα. In trans-presentation or cluster signaling, IL-6/IL-6Rα displayed on one cell activates gp130 on a neighboring recipient cell. Together, these signaling modes expand the spatial and cellular reach of STAT3 activation within the tumor microenvironment. Created in BioRender. Cheng, X. (2026) https://BioRender.com/ytfqqke.

Beyond this classical pathway, IL-6 can also activate STAT3 through trans-signaling. In this setting, IL-6 first binds soluble IL-6Rα (sIL-6Rα), which is generated in part through ADAM17-mediated shedding of membrane-bound IL-6Rα, and the resulting complex can stimulate cells that express gp130 but lack membrane-bound IL-6Rα, including endothelial cells and selected immune populations (9). This expands the range of STAT3-responsive cells and allows inflammatory signals to propagate across distinct tissue compartments. IL-6 additionally uses a third mode of signaling, termed trans-presentation or cluster signaling, in which IL-6 bound to IL-6Rα on one cell is presented to gp130 on an adjacent recipient cell. For example, dendritic cells (DCs) can sense IL-6/IL-6Rα complexes displayed by T cells, thereby activating STAT3 and promoting pathogenic Th17 differentiation (9). These signaling modes highlight a key conceptual point: STAT3 activation is not confined to a single ligand-receptor pair within an individual cell, but can instead be distributed across the tumor ecosystem (**Figure 2**).

**Figure 3:**
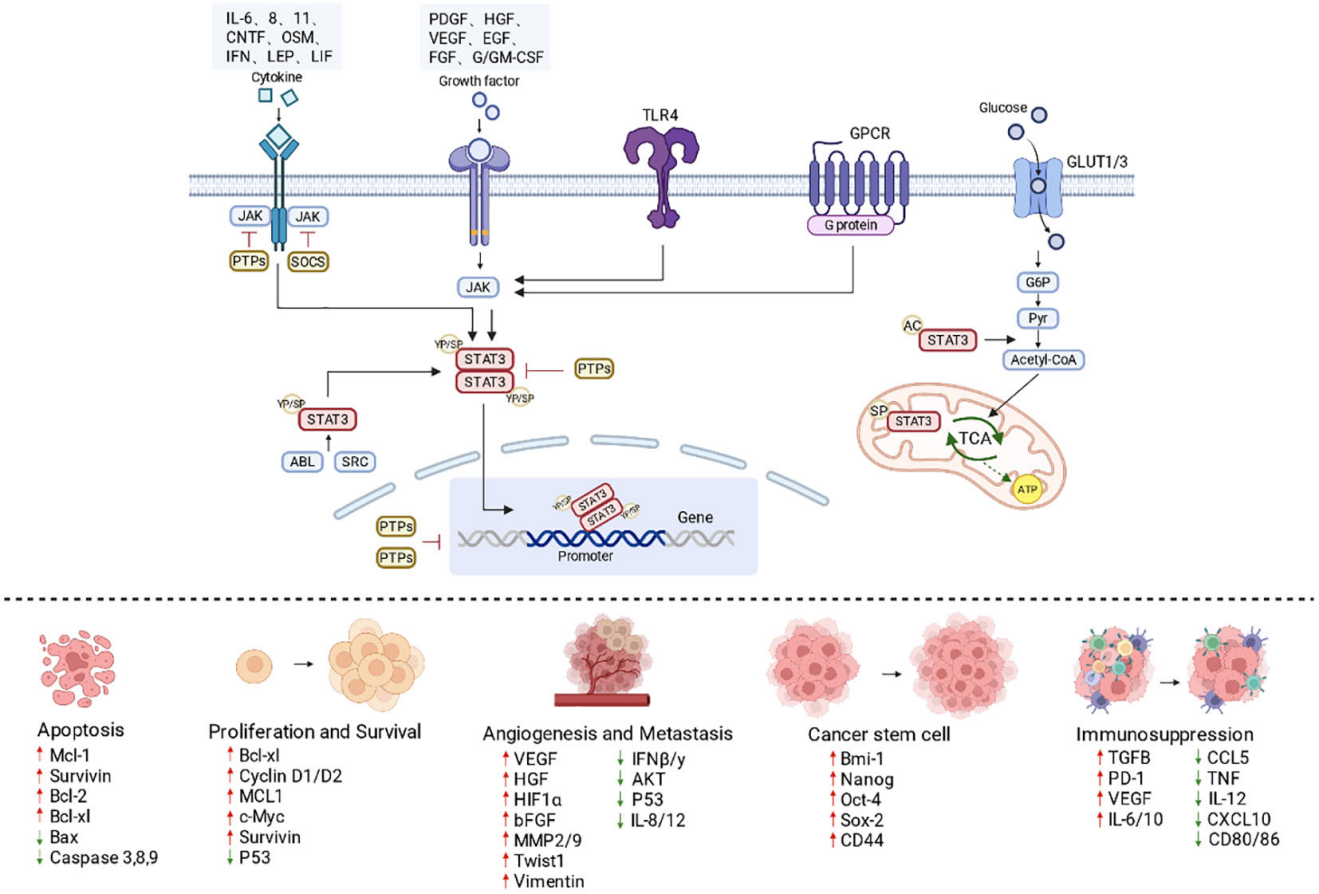
Major downstream consequences of STAT3 activation relevant to tumor immune escape. Cytokines, growth factors, and inflammatory signals induce STAT3 phosphorylation and nuclear translocation through kinases such as JAK, enabling transcriptional programs that promote proliferation and survival, inhibit apoptosis, sustain stemness, drive angiogenesis and metastasis, and establish immunosuppression. Negative regulators such as PTPs and SOCS constrain excessive activation, whereas mitochondrial STAT3 may additionally support metabolic rewiring and energy supply in tumor cells. Created in BioRender. Cheng, X. (2026) https://BioRender.com/ytfqqke.

Once activated, STAT3 functions as a nuclear signal transducer and transcriptional activator. It binds promoters of downstream target genes and coordinates transcriptional networks governing cell-cycle progression, programmed cell death, cellular metabolism, and immune responses. STAT3 promotes proliferation by upregulating Cyclin D1 and c-Myc, thereby facilitating G1/S transition and supporting rapid tumor expansion (10). At the same time, it induces anti-apoptotic genes such as BCL-2, BCL-XL, BCL-W, MCL-1, and Survivin, reducing tumor cell death and prolonging the survival of immunosuppressive cell populations, including M2-like macrophages (10–12). STAT3 also links oncogenic signaling to metabolic and immune adaptation. It enhances glycolytic activity and upregulates molecules such as GLUT1 and B7-H5, helping tumor cells meet bioenergetic demands while simultaneously impairing antitumor immunity (7, 13, 14). Through induction of cytokines and soluble mediators including TGF-β1, VEGF, IL-6, and IL-10, STAT3 further shapes the tumor immune microenvironment by suppressing effector immune cells and promoting the expansion of regulatory T cells (Tregs), thus favoring immune escape (15–18). In addition, STAT3 sustains stemness-associated transcriptional programs, including Oct4 and Sox2, thereby maintaining tumor cell plasticity, migratory potential, and treatment resistance (**Figure 3**) (19).

During tumorigenesis, STAT3 rarely acts in isolation. Instead, it is embedded in a broader signaling architecture in which synergistic and antagonistic interactions determine cellular output. Pro-tumorigenic pathways such as AP-1, Wnt/β-catenin, NF-κB, and VEGF-A cooperate with STAT3 to enhance proliferation, invasion, angiogenesis, anti-apoptotic signaling, and immune escape (20–23). Conversely, antagonistic relationships can also be biologically meaningful. For example, STAT5 signaling promotes mammary epithelial differentiation and constrains proliferation, whereas STAT3 promotes uncontrolled growth and suppresses differentiation. In some contexts, inhibition of STAT5 directly contributes to constitutive STAT3 activation. These interactions help explain why STAT3 can function as a bottleneck rather than a simple linear pathway: its biological output depends on cell identity, signal strength, and pathway crosstalk.

## 3 Tumor-cell-intrinsic STAT3 programs that enable immune escape

A central feature of STAT3-driven immune escape is that it begins within the tumor cell itself. By simultaneously reducing tumor visibility, increasing tumor fitness, and exporting immunosuppressive signals, tumor-intrinsic STAT3 activity establishes the first layer of immune resistance (24–26).

STAT3 promotes immune escape in part by stabilizing a stem-like, therapy-resistant tumor state. Cancer stem cells (CSCs) are a functionally distinct tumor subpopulation characterized by self-renewal, multipotency, relapse potential, and resistance to therapy. These properties also confer an advantage under immune selection, because stem-like tumor cells are more adaptable and less vulnerable to elimination. STAT3 sustains this phenotype by regulating stemness-associated gene expression (27). For example, aryl hydrocarbon receptor (AhR) signaling activates JAK/STAT3 and enhances the transcription of OCT4 and SOX2, forming an “AhR-JAK/STAT3-stemness factor” axis that preserves CSC traits (28). In lung cancer, EGFR signaling cooperates with ERK1/2 and STAT3 to induce SALL4, further maintaining tumor cell stemness (29). Cytokines such as IL-6 and IL-10 released by TAMs also activate STAT3 and directly upregulate SOX2, ALDH, and CD44, thereby linking microenvironmental inflammation to CSC maintenance (30). In glioblastoma (GBM), persistent STAT3 activation enhances Notch/Wnt signaling and induces O6-methylguanine-DNA methyltransferase (MGMT), sustaining glioma stem cell activity and promoting resistance to radiotherapy and chemotherapy (31). These observations indicate that STAT3 does not merely enhance proliferation; it stabilizes a tumor cell state that is intrinsically difficult to eradicate.

A second major mechanism is checkpoint-dominant suppression through PD-L1 regulation. Elevated PD-L1 expression on tumor cells suppresses T cell activation and expansion by engaging PD-1, thereby weakening antitumor immunity and facilitating immune escape. STAT3 controls PD-L1 through both direct and indirect mechanisms and does so in a highly context-dependent manner (10, 32–50). At the direct level, p-STAT3 can bind the PD-L1 promoter and activate its transcription. In triple-negative breast cancer, p-STAT3 and p-STAT1 heterodimers bind the PD-L1 promoter after nuclear translocation, promoting transcription (32). More broadly, the PD-L1 promoter contains STAT3-responsive elements, allowing nuclear p-STAT3 to stimulate PD-L1 expression directly (10, 32, 40). STAT3 can also cooperate with other transcriptional regulators in a tumor-specific manner. In colorectal cancer (CRC), STAT3 and IRF1 co-occupy the PD-L1 promoter, whereas in liver cancer, reciprocal activation between STAT3 and NF-κB generates a transcriptional complex that enhances PD-L1 expression (51–54).

STAT3 also regulates PD-L1 through a multilayered set of indirect mechanisms. It can inhibit PD-L1 degradation by upregulating deubiquitinating enzymes and associated complexes. For example, a CCL5-induced p65/STAT3 complex increases CSN5 expression, thereby blocking proteasomal degradation of PD-L1 (55). STAT3 also upregulates Jun activation domain-binding protein 1 (Jab1), reducing PD-L1 ubiquitination (56), and induces CYLD lysine 63 deubiquitinase (CYLD), which stabilizes the autophagy receptor P62 and indirectly suppresses autophagic PD-L1 degradation (57). At the level of transcriptional intermediates, IL-8 secreted by gastric cancer-associated mesenchymal stem cells activates STAT3 and mTOR to induce c-Myc, which in turn increases PD-L1 expression (58). In ALK-positive anaplastic large-cell lymphoma, STAT3 activates basic leucine zipper ATF-like transcription factor 3 (BATF3), which cooperates with IRF4 to enhance PD-L1 transcription (59). Under hypoxic conditions, STAT3 can also stabilize or induce HIF-1α, further promoting PD-L1 expression (60). STAT3 also reinforces PD-L1 expression by reshaping the immune microenvironment and non-coding RNA networks. STAT3-mediated recruitment and activation of MDSCs increases the release of Arg-1, IL-10, and TGF-β, thereby reinforcing immunosuppression and indirectly sustaining PD-L1 (61–63). In non-small cell lung cancer, STAT3 suppresses miR-197, relieving post-transcriptional repression of PD-L1 and allowing its expression to rise. Together, these findings show that STAT3 does not regulate PD-L1 through one isolated route; rather, it builds a robust, redundant checkpoint program at transcriptional, post-translational, and ecosystem levels.

STAT3 further enables tumor cells to export immunosuppressive signals into the tumor microenvironment (TME). One prominent example is indoleamine 2, 3-dioxygenase 1 (IDO1). Activated STAT3 increases IDO expression and maintains IDO1 activity, thereby driving extensive tryptophan catabolism in the TME (19, 64–66). Local tryptophan depletion directly impairs activation and differentiation of effector T cells, including CD8+ T cells, whereas kynurenine and other metabolites suppress T cell function and promote MDSC development. These MDSCs further inhibit T cell responses and enhance Treg differentiation, reinforcing immune tolerance (19, 64–66). Beyond IDO1, STAT3 also induces a broader immunosuppressive secretome. Activated STAT3 upregulates IL-10, IL-6, TGF-β, and VEGF, which promote PGE2 and COX-2 signaling, inhibit DC maturation and cytotoxic lymphocyte function, and support the formation of an immunosuppressive microenvironment (15, 18, 25). Thus, STAT3 endows tumor cells not only with resistance to immune attack, but with the ability to reshape surrounding immune populations in ways that propagate suppression. These findings position STAT3 not only as a mediator of tumor cell survival, but also as an architect of the suppressive microenvironment.

A further layer of immune escape arises from reduced tumor immunogenicity and enhanced survival. Persistent STAT3 activation can downregulate MHC class I and II expression, impair antigen processing and presentation, and thereby diminish T cell recognition (7, 30, 67, 68). In addition, by increasing MHC class I expression in a way that favors engagement of NK-cell inhibitory receptors such as killer-cell immunoglobulin-like receptors (KIR) and Ly49, STAT3 may also reduce NK-cell cytotoxicity (18). In parallel, STAT3 protects tumor cells from apoptosis. It directly induces anti-apoptotic genes such as Survivin and upregulates oncogenic survival factors including BCL-2, c-Myc, and Cyclin D1 (10, 11, 69). In Hodgkin lymphoma, STAT3 enhances Hodgkin and Reed-Sternberg (HRS) cell survival by promoting IL-6-dependent induction of MCL1 (70). STAT3 also interacts with protein kinase R (PKR), inhibiting its activity and blocking endoplasmic reticulum stress-induced apoptosis (71). By suppressing caspase activation and decreasing the release of immunogenic death signals, STAT3 reduces both tumor cell death and the immunological consequences of that death (45, 70–73). Thus, STAT3 not only promotes tumor cell survival, but also preserves that survival in a state less likely to elicit productive immunity.

**Figure 4:**
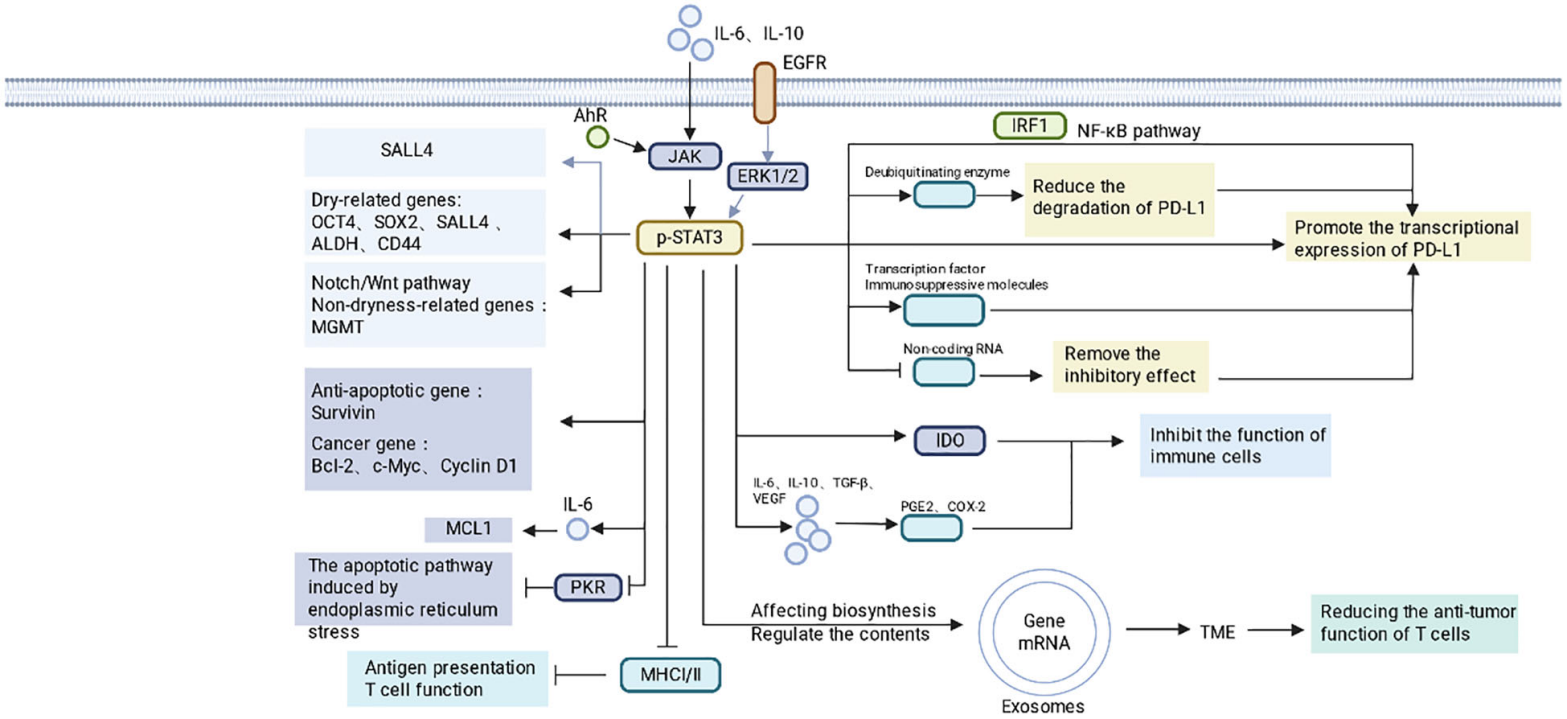
Tumor-cell-intrinsic mechanisms by which STAT3 drives immune escape. Activated STAT3 sustains stemness programs through factors such as SALL4, OCT4, and SOX2; enhances survival through BCL-2 family members, Survivin, c-Myc, and related anti-apoptotic networks; promotes checkpoint-dominant suppression by transcriptionally and post-transcriptionally increasing PD-L1; reduces immunogenicity by impairing antigen presentation; and reshapes the microenvironment through immunosuppressive secreted factors and exosome-mediated signaling. Created in BioRender. Cheng, X. (2026) https://BioRender.com/ytfqqke.

STAT3-mediated immune escape extends beyond soluble factors to extracellular vesicle communication. STAT3 regulates both exosome biogenesis and the composition of exosomal cargo (37, 74–76). Tumor cell-derived exosomes can be loaded with immunosuppressive molecules such as B7-H4-related transcripts or specific miRNAs and released into the TME, where they impair T cell responses (74, 75). Notably, exosomes can also feed back to activate STAT3 in recipient cells, creating a reciprocal loop between vesicle-mediated communication and STAT3 signaling (6, 77, 78). This mechanism further illustrates the systems-level nature of STAT3-driven immune escape: the pathway does not remain confined to the cell in which it is activated, but broadcasts immune-regulatory information across the tumor ecosystem (**Figure 4**).

## 4 Immune-cell-intrinsic STAT3 programs that suppress antitumor immunity

While tumor-cell-intrinsic STAT3 helps malignant cells evade recognition and killing, immune-cell-intrinsic STAT3 activity rewires the surrounding microenvironment into a tolerant and suppressive niche. In this way, STAT3 acts on both sides of the tumor-immune interface (25, 26).

A key consequence of aberrant STAT3 activation in the TME is the suppression of effector T cells together with enhanced differentiation and recruitment of Tregs (56, 67, 70). STAT3 promotes Treg expansion directly and indirectly. Tregs release IL-10 and TGF-β, which inhibit effector T cell activity and reduce the expression of cytotoxic molecules such as perforin and granzymes (72, 79). At the same time, chronic STAT3 activation in tumor cells and stromal compartments increases the production of chemokines such as CCL2 and CCL17 and augments signals including IL-6 and EGFR, thereby recruiting Tregs and M2-like TAMs into the TME and strengthening their suppressive function (80). This layered control shifts the balance from effective tumor rejection toward immune tolerance. STAT3 also amplifies T cell suppression indirectly through myeloid intermediates. By increasing PD-L1 or IDO1 expression and enhancing MDSC recruitment, STAT3 creates an environment in which T cell proliferation and effector function are persistently restrained (19, 66, 81). In addition, STAT3-driven M2 macrophage activation can secondarily influence Treg and effector T cell behavior, further consolidating immunosuppression (82). Thus, STAT3-mediated T cell dysfunction is not confined to T cell-intrinsic signaling, but reflects an ecosystem-wide restructuring of suppressive inputs.

Beyond promoting Treg-rich ecosystems, STAT3 also suppresses antitumor immunity through cell-intrinsic effects in CD8^+^ T cells. Inhibition of STAT3 enhances the *in vivo* expansion, tumor infiltration, and antitumor activity of adoptively transferred CD8^+^ T cells (83). STAT3 signaling in CD8^+^ T cells can also limit tumor accumulation by restraining IFNγ-dependent CXCR3/CXCL10 trafficking programs (84). Mechanistically, activated STAT3 promotes fatty acid oxidation in CD8^+^ effector T cells and thereby contributes to a metabolically dysfunctional or exhaustion-like state in tumors (85). In parallel, STAT3-dependent transcriptional circuitry suppresses cytotoxic gene expression, further weakening durable CD8^+^ T-cell effector function (86).

STAT3 is also a central regulator of MDSC development, accumulation, and function (11, 87). Sustained STAT3 activation drives the expansion of immature myeloid populations and supports their differentiation into MDSCs (80, 88). Once established, these cells secrete immunosuppressive mediators such as arginase, IDO-related factors, and other inhibitory molecules that blunt T and NK cell activity (6, 61–63, 89). They can also enhance Th17-associated inflammatory programs, which in some settings further promote tumor progression and immune dysfunction. Importantly, MDSCs are not only effectors of immunosuppression but also amplifiers of the STAT3 program. Their recruitment and survival reinforce the suppressive microenvironment, leading to a self-sustaining myeloid reservoir that continuously undermines antitumor immunity. In this sense, STAT3-dependent MDSC biology represents one of the major bridges between chronic inflammation and durable immune resistance.

Macrophage polarization provides another major immune-cell-intrinsic route through which STAT3 promotes tumor escape. STAT3 activation suppresses M1-like inflammatory macrophage functions while promoting polarization of TAMs toward an M2-like phenotype (6, 11, 31, 80, 90, 91). M2-type macrophages produce IL-10, TGF-β, and other anti-inflammatory mediators that dampen antitumor immunity, support tissue remodeling, and promote angiogenesis. In addition, they can express PD-L1 and directly inhibit T cell function (92). STAT3-mediated M2 polarization occurs through both direct transcriptional control and indirect communication with tumor cells. In lung cancer, activated STAT3 induces tumor-derived CCL2 and CCL17, which recruit M2-type TAMs and enhance their suppressive function (69). In colorectal cancer, VSIG4 activates JAK2/STAT3 signaling, and p-STAT3 induces PPAR-γ expression, fueling M2 polarization through fatty acid oxidation (93). This metabolic component is especially important because it indicates that STAT3 not only specifies macrophage phenotype but also stabilizes that phenotype through bioenergetic rewiring.

STAT3 also weakens antitumor immunity by disrupting antigen presentation. This effect is especially evident in DCs, where STAT3 suppresses maturation, differentiation, and cytokine production, thereby reducing antigen-presenting capacity and impairing immune signaling (11, 61). At the molecular level, STAT3 downregulates surface HLA-I molecules and antigen transporters such as TAP1/2, undermining the machinery required for effective antigen presentation (16). The consequence is a failure to prime and sustain T cell responses, which allows tumor cells to escape recognition even when antigenic material is present.

NK cells represent another important cell-intrinsic target of STAT3-mediated immunosuppression. NK-cell-specific loss of Stat3 enhances NK-cell-dependent tumor surveillance and increases the expression of perforin, granzyme B, and DNAM-1, indicating that persistent STAT3 signaling restrains the core cytolytic machinery of NK cells (94). In human tumor settings, tumor-derived IL-6 and IL-8 can activate STAT3 in NK cells and blunt NK-cell function, further limiting innate immune pressure on malignant cells (95).

An additional, less frequently discussed STAT3-dependent suppressive axis involves regulatory B cells (Bregs). B-cell-intrinsic STAT3 activation is required for inducible IL-10 production downstream of TLR signaling, providing a mechanistic basis for the generation of immunosuppressive IL-10-producing B cells (96). In tumors, B cells with activated STAT3 have been shown to promote tumor progression, at least in part through pro-angiogenic activity (97). Tumor-evoked regulatory B cells can also promote metastasis by converting resting CD4^+^ T cells into FoxP3^+^ Tregs in a TGF-β-dependent manner (98). Together, these findings support inclusion of Bregs in the STAT3-centered immunosuppressive network, although the tumor-specific differentiation circuitry remains less completely defined than that described for MDSCs or TAMs.

**Figure 5:**
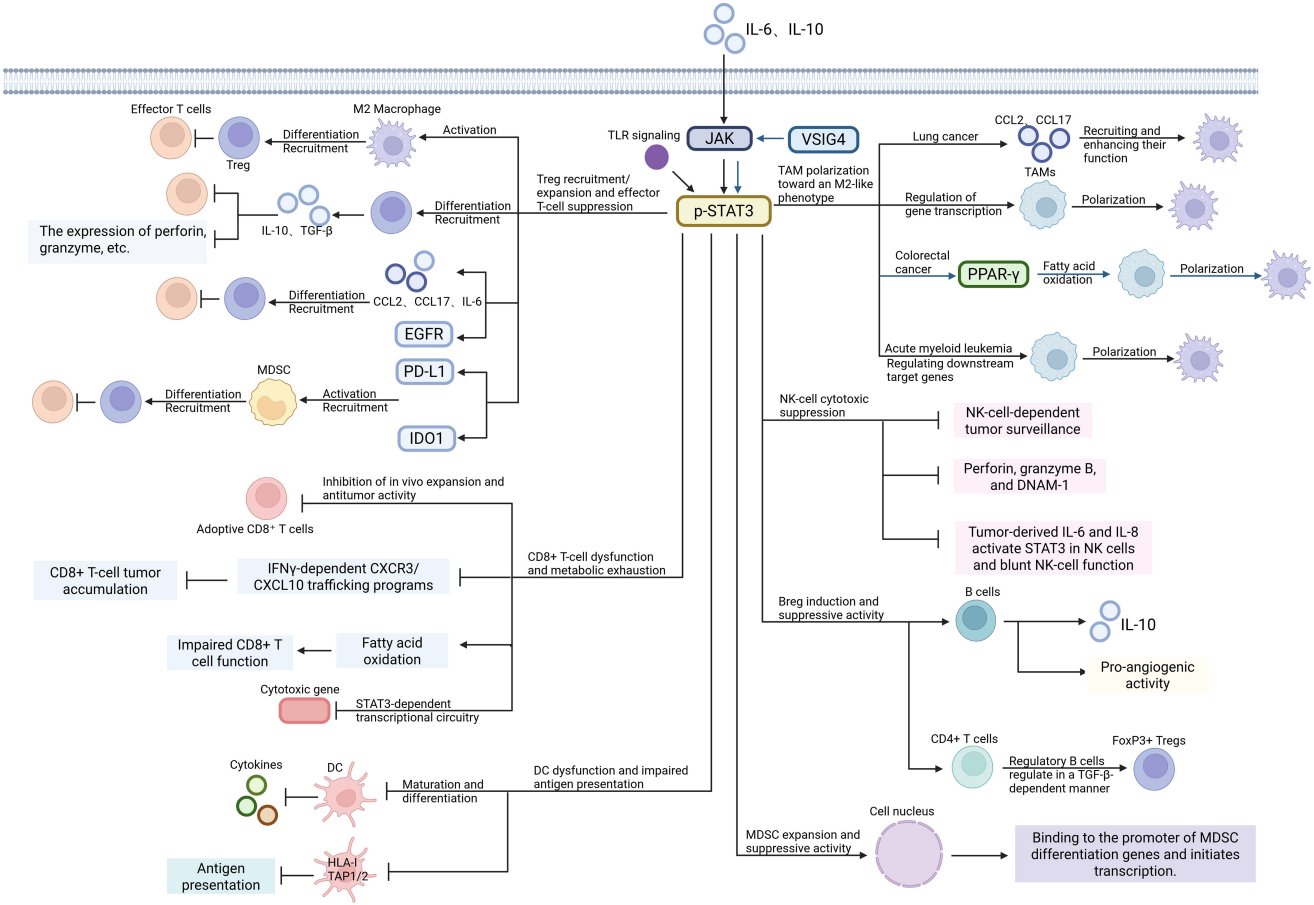
Immune-cell-intrinsic effects of STAT3 that establish an immunosuppressive microenvironment. STAT3 activation promotes Treg differentiation, expands MDSCs, drives TAM polarization toward an M2-like phenotype, impairs DC maturation and antigen presentation, and suppresses CD8^+^ T-cell, NK-cell, and regulatory B-cell-associated antitumor immunity. Through these combined effects, STAT3 weakens effector immunity and stabilizes a tumor-permissive immune niche. Created in BioRender. Cheng, X. (2026) https://BioRender.com/ytfqqke.

Taken together, these immune-cell-intrinsic mechanisms establish STAT3 as a systems-level regulator of immunosuppressive cell fate within the tumor microenvironment. Beyond Treg dominance, myeloid suppression, macrophage polarization, and APC paralysis, STAT3 also restrains CD8^+^ T-cell fitness, suppresses NK-cell cytotoxicity, and supports regulatory B-cell programs, thereby coordinating a broader multilayered network of immune escape (**Figure 5**, **Table 1**).

**Table 1:** Tumor-cell-intrinsic and immune-cell-intrinsic mechanisms by which STAT3 drives immune escape.

Cellular compartment	Mechanistic category	Representative mechanisms	References
Tumor cells	Maintenance of stemness and tumor plasticity	Activation of the AhR promotes the transcriptional expression of stemness genes, such as OCT4 and SOX2, via the JAK/STAT3 pathway	(28)
Tumor cells	Maintenance of stemness and tumor plasticity	In lung cancer, EGFR directly regulates the STAT3 signaling pathway and cooperates with the ERK1/2 signaling pathway to induce the expression of the stem cell regulatory factor SALL4	(14)
Tumor cells	Maintenance of stemness and tumor plasticity	Cytokines (IL-6/IL-10) secreted by TAMs activate the STAT3 pathway, which directly regulates the expression of CSCs-associated stemness genes, such as SOX2, ALDH, and CD44	(30)
Tumor cells	Maintenance of stemness and tumor plasticity	STAT3 activation enhances Notch/Wnt pathway signaling and promotes overexpression of the non-stemness gene MGMT	(31)
Tumor cells	PD-L1 upregulation and checkpoint-dominant suppression	STAT3 translocates directly into the nucleus, binds to the PD-L1 promoter, and promotes its transcriptional expression	(10, 33–50)
Tumor cells	PD-L1 upregulation and checkpoint-dominant suppression	In colorectal and liver cancers, STAT3 can synergize with pathways such as IRF1 or NF-κB to directly bind to the promoter region of PD-L1, thereby promoting its transcriptional expression	(51–53)
Tumor cells	PD-L1 upregulation and checkpoint-dominant suppression	STAT3 upregulates the transcription of deubiquitinating enzymes and associated complexes, which indirectly reduces PD-L1 degradation and consequently increases its expression	(55–57)
Tumor cells	STAT3 pathway promotes immune escape by upregulating PD-L1 expression	STAT3 promotes the expression of transcription factors or complexes, which in turn indirectly upregulate the transcription of PD-L1	(54, 58–60)
Tumor cells	PD-L1 upregulation and checkpoint-dominant suppression	STAT3 facilitates the recruitment of immunosuppressive cells and the release of immunosuppressive molecules, thereby indirectly upregulating the expression of PD-L1	(61–63)
Tumor cells	PD-L1 upregulation and checkpoint-dominant suppression	STAT3 regulates non-coding RNAs and suppresses their expression, thereby alleviating their inhibitory effect on PD-L1 and indirectly promoting PD-L1 expression	(42)
Tumor cells	Induction of an immunosuppressive secretome	Activated STAT3 can upregulate the expression of IDO in tumor cells, thereby suppressing the function of immune cells and promoting immune escape	(19, 64–66)
Tumor cells	Induction of an immunosuppressive secretome	Activated STAT3 upregulates IL-10, IL-6, TGF-β, and VEGF, which in turn elevate PGE2 and COX-2, thereby suppressing immune cell function and establishing an immunosuppressive microenvironment	(6, 11, 15–17, 25, 30, 32, 52, 60, 61, 67, 69, 80, 99–102)
Tumor cells	Impaired antigen presentation and reduced immunogenicity	Persistent activation of STAT3 can downregulate the expression of MHC class I/II molecules, leading to a reduction in antigen presentation and the suppression of T cell function	(7, 18, 30, 67, 68)
Tumor cells	Apoptosis resistance and survival advantage	STAT3 can directly induce the expression of the classical anti-apoptotic gene Survivin, thereby inhibiting apoptosis in tumor cells	(11)
Tumor cells	Apoptosis resistance and survival advantage	As a central transcriptional regulator, STAT3 upregulates the expression of multiple oncogenes, including BCL-2, c-Myc, and Cyclin D1, thereby directly or indirectly promoting the proliferation and survival of tumor cells	(10, 69)
Tumor cells	Apoptosis resistance and survival advantage	In Hodgkin lymphoma, STAT3 can enhance the anti-apoptotic capacity of HRS cells by upregulating IL-6, which promotes anti-apoptosis-related genes such as MCL1	(70)
Tumor cells	Apoptosis resistance and survival advantage	STAT3 interacts with PKR, inhibiting its enzyme activity and thereby blocking the endoplasmic reticulum stress-induced apoptotic pathway	(71)
Tumor cells	Exosome-mediated immunosuppressive signaling	STAT3 regulates the exosomal cargo, serving as a vehicle to load and release specific genes (such as B7-H4) or miRNAs into the TME. These exosomal contents interact with T cells, thereby impairing the anti-tumor immune response of the host	(74, 75)
Tumor cells	Exosome-mediated immunosuppressive signaling	STAT3 influences the biogenesis of exosomes	(37)
Immune cells	Treg recruitment/expansion and effector T-cell suppression	STAT3 promotes the differentiation and recruitment of Tregs. The anti-inflammatory cytokines, such as IL-10 and TGF-β, secreted by Tregs themselves can further suppress the activity of effector T cells and the expression of cytotoxic molecules, including perforin and granzymes	(72, 79)
Immune cells	Treg recruitment/expansion and effector T-cell suppression	STAT3 activation promotes the secretion of chemokines such as CCL2 and CCL17, while upregulating the expression of IL-6 and EGFR, thereby mediating the recruitment of Tregs and directly inhibiting effector T cells	(80)
Immune cells	Treg recruitment/expansion and effector T-cell suppression	STAT3 enhances the activation and recruitment of MDSCs by upregulating PD-L1 expression or promoting the transcription and protein expression of IDO1, thereby indirectly suppressing T cell proliferation and function. Additionally, it further augments the differentiation and activity of Tregs	(11, 19, 66, 81)
Immune cells	Treg recruitment/expansion and effector T-cell suppression	STAT3-mediated activation of M2-type macrophages can also indirectly regulate the functions of Tregs and effector T cells	(82)
Immune cells	CD8+ T-cell dysfunction and metabolic exhaustion	Inhibition of STAT3 enhances the *in vivo* expansion, tumor infiltration, and antitumor activity of adoptively transferred CD8+ T cells	(83)
Immune cells	CD8+ T-cell dysfunction and metabolic exhaustion	STAT3 signaling restrains IFNγ-dependent CXCR3/CXCL10 trafficking programs, thereby limiting CD8+ T-cell tumor accumulation	(84)
Immune cells	CD8+ T-cell dysfunction and metabolic exhaustion	Activated STAT3 promotes fatty acid oxidation and contributes to a metabolically dysfunctional or exhaustion-like state in CD8+ effector T cells; in parallel, STAT3-dependent transcriptional circuitry suppresses cytotoxic gene expression	(85, 86)
Immune cells	MDSC expansion and suppressive activity	Activated STAT3 (p-STAT3) translocates into the nucleus, where it directly binds to the promoters of genes involved in MDSC differentiation and initiates their transcription	(6, 61–63, 80, 88, 89)
Immune cells	TAM polarization toward an M2-like phenotype	STAT3 induces the polarization of TAMs toward the M2 phenotype through direct regulation of gene transcription	(6, 11, 31, 80, 90, 91)
Immune cells	TAM polarization toward an M2-like phenotype	STAT3 activation induces lung cancer cells to secrete chemokines such as CCL2 and CCL17, which recruit M2-type TAMs to the tumor site and enhance their function	(69)
Immune cells	TAM polarization toward an M2-like phenotype	In acute myeloid leukemia, activated STAT3 regulates downstream target genes, inducing the polarization of macrophages toward the M2 phenotype	(92)
Immune cells	TAM polarization toward an M2-like phenotype	In colorectal cancer, VSIG4 activates JAK2/STAT3, and p-STAT3 induces PPAR-γ expression, which fuels M2 polarization through fatty acid oxidation	(93)
Immune cells	DC dysfunction and impaired antigen presentation	STAT3 impedes its maturation and differentiation, thereby attenuating its antigen-presenting potential, and also disrupts the normal secretion of cytokines	(11, 61)
Immune cells	DC dysfunction and impaired antigen presentation	STAT3 directly undermines the molecular basis of antigen presentation by downregulating the expression of HLA-I molecules on the surface of DCs and the antigen transport-associated proteins TAP1/2	(16)
Immune cells	NK-cell cytotoxic suppression	NK-cell-specific loss of Stat3 enhances NK-cell-dependent tumor surveillance and increases the expression of perforin, granzyme B, and DNAM-1, indicating that persistent STAT3 signaling restrains the core cytolytic machinery of NK cells	(94)
Immune cells	NK-cell cytotoxic suppression	Tumor-derived IL-6 and IL-8 activate STAT3 in NK cells and blunt NK-cell function, thereby limiting innate immune pressure on malignant cells	(95)
Immune cells	Breg induction and suppressive activity	B-cell-intrinsic STAT3 activation is required for inducible IL-10 production downstream of TLR signaling, providing a mechanistic basis for the generation of immunosuppressive IL-10-producing B cells	(96)
Immune cells	Breg induction and suppressive activity	B cells with activated STAT3 promote tumor progression, at least in part through pro-angiogenic activity	(97)
Immune cells	Breg induction and suppressive activity	Tumor-evoked regulatory B cells can promote metastasis by converting resting CD4+ T cells into FoxP3+ Tregs in a TGF-β-dependent manner	(98)

## 5 STAT3-driven metabolic and multicellular ecosystems of immune escape

The immunosuppressive role of STAT3 cannot be fully understood by analyzing isolated cell types alone. A defining feature of STAT3 biology is its ability to organize multicellular and metabolic circuits that stabilize immune resistance across the tumor ecosystem.

In tumor cells, IL-6/JAK/STAT3 signaling promotes multiple forms of metabolic rewiring, including increased aerobic glycolysis, lipogenesis, and glutamine metabolism (103, 104). These changes provide ATP and biosynthetic intermediates required for rapid proliferation while helping tumor cells withstand oxidative and nutrient stress. STAT3 transcriptionally activates HIF-1α and hexokinase 2 (HK2), thereby promoting glycolytic enzyme expression and glucose flux (103). It can also translocate to mitochondria in post-translationally modified forms, where it enhances mitochondrial complex I activity, reduces ROS release, and helps maintain redox homeostasis, survival, and proliferation (103). Through crosstalk with MAPK and PI3K/AKT, STAT3 further integrates metabolic remodeling with invasive behavior and treatment resistance (104). In addition, emerging evidence suggests that this metabolic program is closely coupled to lactate production. Activated STAT3 can sustain LDHA-centered glycolysis and thereby increase lactate output (105, 106), whereas lactate-rich conditions can in turn enhance STAT3 phosphorylation and nuclear accumulation, indicating a feed-forward interaction between STAT3 activity and lactate-driven metabolic remodeling (107).

This metabolic role of STAT3 is increasingly supported across multiple tumor contexts. Examples from specific tumor contexts reinforce this point. In NSCLC, IL-10RA overexpression activates STAT3 and increases expression of glycolytic enzymes such as GLUT3, PKM2, HK2, and PFKL, promoting lactate production and an enhanced glycolytic phenotype (108). At the same time, STAT3 increases expression of CPT1A, ACADM, and PPAR-γ, thereby promoting fatty acid oxidation and ATP generation (108). In HCC, PLOD1 activates an NF-κB/IL-6/STAT3 axis that enhances pyruvate dehydrogenase activity and rebalances key TCA intermediates, supporting growth, stemness, and anti-apoptotic capacity (109). Beyond these enzyme-level changes, lactate-derived lysine lactylation adds an epigenetic layer to STAT3-centered metabolic regulation. Histone lactylation, including H3K18la, has been linked to active chromatin states and increased chromatin accessibility, suggesting that lactylation can fine-tune the transcriptional context in which STAT3 operates (110–112). Recent work further indicates that H3K18la-associated chromatin remodeling may help stabilize tumor-promoting transcriptional programs relevant to immune escape (110). These observations indicate that STAT3 functions as a metabolic gatekeeper of tumor persistence under immune and therapeutic stress.

STAT3-dependent metabolic reprogramming is not restricted to tumor cells. It also shapes the metabolic state of immune populations, thereby influencing their differentiation and function. In CRC, tumor-derived lactate increases VSIG4 expression in macrophages and activates JAK2/STAT3 signaling, which induces PPAR-γ and fatty acid oxidation, driving macrophages toward an M2 phenotype (93). These polarized macrophages then secrete heparin-binding EGF-like growth factor, promote CRC proliferation and invasion, suppress apoptosis, and reduce CD8^+^ T cell infiltration (93). Similarly, tumor-derived cytokines induced by PKM2 can activate STAT3 in MDSCs, increasing cathepsin expression and suppressing T cell migration, adhesion, and survival (113). Importantly, additional evidence indicates that lactate/lactylation can reinforce this immune-suppressive circuitry more directly. In breast cancer, tumor-derived lactate activates ERK/STAT3 signaling to drive M2 macrophage polarization (114). In colorectal cancer-associated macrophages, lactate-driven histone lactylation suppresses RARγ expression and activates a TRAF6-IL-6-STAT3 signaling cascade, further promoting a tumor-supportive macrophage state (115). These findings are important because they show that STAT3 not only responds to metabolic cues but also converts them into stable immune suppressive phenotypes. The resulting metabolic states are not passive correlates of immune dysfunction; they are active determinants of immune escape.

Another distinctive feature of STAT3 is its ability to operate across tissues. In pancreatic ductal adenocarcinoma (PDAC), tumor-derived IL-6 activates STAT3 in hepatocytes, leading to metabolic reprogramming that suppresses hepatic β-oxidation and ketogenesis and contributes to early cachexia (116). This systemic metabolic disruption weakens the host and indirectly exacerbates immunosuppression. Notably, in some settings tumor cells do not directly produce IL-6; rather, immune cells within the TME provide the cytokine that initiates STAT3-mediated metabolic rewiring (116). This illustrates how STAT3 signaling can emerge from multicellular cooperation rather than a tumor-cell-autonomous process.

More broadly, STAT3-dependent metabolic rewiring creates a self-reinforcing tumor–immune regulatory loop. Metabolically adapted tumor cells proliferate, survive, and metastasize more efficiently while releasing cytokines such as IL-6 that activate STAT3 in immune cells. These immune cells then acquire suppressive phenotypes and produce additional factors that sustain STAT3 activation in tumor cells. Lactate and lactylation appear to strengthen this reciprocity by linking tumor glycolysis to epigenetic remodeling and sustained STAT3 signaling across both tumor and immune compartments. Through this reciprocal signaling, metabolic adaptation and immune escape become coupled processes rather than separate hallmarks. This reciprocal circuitry may also explain why disrupting STAT3 could simultaneously impair tumor fitness and relieve immune suppression.

## 6 Therapeutic exploitation of the STAT3 axis

Because STAT3 coordinates tumor survival, immune suppression, and metabolic adaptation, it represents a highly attractive therapeutic target. At the same time, its centrality within tumor and immune signaling networks makes it challenging to inhibit effectively. The therapeutic question is therefore not simply whether STAT3 can be targeted, but how it should be targeted in a biologically rational and clinically actionable manner. As discussed above, STAT3 exerts both tumor-intrinsic and immune-cell-intrinsic immunosuppressive effects, suggesting that STAT3-targeted therapy may be particularly valuable in combination with immunotherapy to enhance antitumor responses and reduce resistance. Efforts to disrupt this pathway have generated a diverse therapeutic landscape spanning small molecules, natural compounds, and biotechnology-based approaches.

Current pharmacologic strategies can be broadly divided into indirect and direct approaches. Indirect inhibitors suppress upstream activators of STAT3, including JAK kinases, Src family kinases (SFKs), and receptor tyrosine kinases such as EGFR (13, 117, 118). Representative agents include ruxolitinib, tofacitinib, fedratinib, dasatinib, bosutinib, erlotinib, gefitinib, and afatinib, which reduce STAT3 activation by blocking the signaling context in which it is engaged (13, 89, 117, 118). Direct inhibitors, by contrast, attempt to block STAT3 itself by interfering with phosphorylation, SH2-domain engagement, dimerization, nuclear translocation, or transcriptional activity. Representative compounds include OPB-31121, Stattic, S3I-201, STX-0119, BP-1-102, LLL12B, FLLL32, Bt354, niclosamide, and AZD9150 (52, 53, 119–121). Collectively, these studies support the idea that both upstream blockade and direct inhibition can suppress STAT3 signaling and thereby limit tumor progression and immune escape (**Table 2**).

**Table 2:** Representative small-molecule and kinase-based strategies targeting the STAT3 axis.

Agent	Target level	Representative mechanism	Inhibition type	References	Clinical stage	NCT number
Stattic	Directly targeting STAT3	It inhibits the phosphorylation of STAT3 by binding to its SH2 domain.	Direct Inhibition	(25, 39, 43, 47, 51, 53, 57, 58, 89, 90, 92, 93, 120, 121)	Preclinical studies	None
S3I-201	Directly targeting STAT3	It inhibits transcriptional activity and blocks the interaction with other signaling molecules.	Direct Inhibition	(13, 35)	Preclinical studies	None
STX-0119	Directly targeting STAT3	It inhibits the DNA binding activity of STAT3.	Direct Inhibition	(15, 61)	Preclinical studies	None
BP-1-102	Directly targeting STAT3	Inhibition of STAT3 Tyr705 phosphorylation.	Direct Inhibition	(12, 37)	Preclinical studies	None
LLL12B	Directly targeting STAT3	Suppresses JAK2/STAT3 phosphorylation	Direct Inhibition	(80)	Preclinical studies	None
FLLL32	Directly targeting STAT3	It inhibits the binding of STAT3 to DNA and its phosphorylation.	Direct Inhibition	(15, 87)	Preclinical studies	None
OPB-31121	Directly targeting STAT3	It directly binds to the SH2 domain of STAT3, thereby inhibiting the activation of STAT3.	Direct Inhibition	(11, 15, 18, 40, 52)	Phase I/II	NCT00955812 (I) NCT00511082 (I) NCT00657176 (I) NCT01406574 (I/II)
Bt354	Directly targeting STAT3	It inhibits the phosphorylation of STAT3 by binding to its SH2 domain.	Direct Inhibition	(32)	Preclinical studies	None
Niclosamide	Directly targeting STAT3	It suppresses STAT3 signaling or activation.	Direct Inhibition	(55, 89)	Phase I/II	NCT02687009 (I) NCT02532114 (I) NCT03123978 (I) NCT02519582 (II)
WP1066	JAK inhibitors	It inhibits JAK2/STAT3 signaling and p-STAT3.	Indirect Inhibition	(15, 31, 61, 65, 89, 122, 123)	Phase I/II	NCT04334863 (I) NCT01904123 (I) NCT05879250 (II)
Ruxolitinib	JAK inhibitors	It inhibits the upstream proteins (JAK kinases) of the STAT3-related pathways.	Indirect Inhibition	(11, 13, 15, 16, 41, 62, 89)	Phase I/II/III	NCT06008275 (I) NCT07219576 (Ib/II) NCT06616155 (Ib/II) NCT06991101 (II) NCT02117479 (III)
Tofacitinib	JAK inhibitors	It inhibits the phosphorylation of STAT3 by suppressing the JAK kinase.	Indirect Inhibition	(15, 78)	Phase III	NCT05326464 (III)
Fedratinib	JAK inhibitors	Selectively inhibits JAK2, thereby reducing downstream STAT3/STAT5 signaling.	Indirect Inhibition	(15)	Phase I/II/III	NCT04955938 (I) NCT05393674 (II) NCT03952039 (III)
SD-1029	JAK inhibitors	It inhibits JAK2 and reduces the phosphorylation level of STAT3.	Indirect Inhibition	(13)	Preclinical studies	None
AZ960	JAK inhibitors	It inhibits JAK2 to suppress the STAT3 signaling pathway.	Indirect Inhibition	(25)	Preclinical studies	None
AG490	JAK inhibitors	It inhibits JAK2 to suppress the STAT3 signaling pathway.	Indirect Inhibition	(6, 33, 60, 66, 93, 120)	Preclinical studies	None
AZD1480	JAK inhibitors	It inhibits JAK1/2 to reduce the phosphorylation of STAT3.	Indirect Inhibition	(15, 36, 61, 89, 119, 124)	Phase I (terminated)	NCT01219543 (I, terminated) NCT01112397 (I, terminated)
STM2457	METTL3 inhibitors	It reduces m6A modification of JAK1 mRNA and thereby attenuates JAK1/STAT3 signaling.	Indirect Inhibition	(99)	Preclinical studies	None
Sunitinib	Multitarget RTK inhibitors	A multitarget RTK inhibitor that targets VEGFR, PDGFR, KIT, FLT3, CSF1R, and RET, thereby indirectly attenuating downstream STAT3 signaling.	Indirect Inhibition	(25, 79)	Phase II/III	NCT01621568 (II) NCT00083889 (III) NCT00428597 (III)
Dasatinib	SFK inhibitors	It inhibits the STAT3 signaling pathway by targeting the SRC and ABL kinases.	Indirect Inhibition	(118)	Phase I/II	NCT05198843 (Ib/II) NCT05036226 (I/II)
Bosutinib	SFK inhibitors	It inhibits the STAT3 pathway by suppressing the upstream kinase SRC.	Indirect Inhibition	(118)	Phase I/II	NCT03854903 (I) NCT00959946 (I/II)
Erlotinib	EGFR inhibitors	It inhibits the indirect action of upstream EGFR on STAT3.	Indirect Inhibition	(89)	Phase II/III	NCT00600587 (II) NCT01667562 (IIIb) NCT02193282 (III)
Gefitinib	EGFR inhibitors	It inhibits the indirect action of upstream EGFR on STAT3.	Indirect Inhibition	(117)	Phase I/II/III	NCT00052208 (I/II) NCT02347839 (II) NCT00322452 (III)
Afatinib	EGFR inhibitors	It inhibits the indirect action of upstream EGFR on STAT3.	Indirect Inhibition	(89)	Phase II/III	NCT00525148 (II) NCT00949650 (III) NCT01121393 (III)

In addition to synthetic compounds, natural products and traditional medicine-derived agents also exhibit STAT3-inhibitory activity. These compounds are notable for their multi-target properties and potentially lower toxicity profiles. For example, cucurbitacin I indirectly suppresses STAT3 by inhibiting upstream kinases such as JAK2 and SFKs, whereas frankincense and myrrh extracts directly reduce p-STAT3 levels in hepatocellular carcinoma cells (25, 102). Other reported agents, including curcumin, osthole, tetrandrine, PZH2108, platycodon grandiflorus, piperlongumine, resveratrol, quercetin, cryptotanshinone, β-elemene, and Yifei Sanjie Formula, further illustrate the diversity of natural-product-based approaches for targeting the STAT3 axis (25, 32, 125) (**Table 3**).

**Table 3:** Natural products and traditional medicine-derived agents reported to inhibit STAT3 signaling.

Agent	Target level	Representative mechanism	Inhibition type	References	Clinical stage	NCT nunber
Curcumin	Directly targeting STAT3 and upstream regulators	It suppresses STAT3 signaling by reducing STAT3 DNA-binding and activation, at least in part through inhibition of upstream JAK1/2 signaling and phosphatase-dependent negative regulation.	Direct and indirect Inhibition	(11, 15, 25, 61, 79, 89)	Phase I/II	NCT01294072 (I) NCT00641147 (II) NCT07248020 (II)
Osthole	Directly targeting STAT3	It inhibits the phosphorylation of STAT3 by binding to its SH2 domain.	Direct Inhibition	(32)	Preclinical studies	None
Tetrandrine	Targeting upstream regulators	It inhibits JAK2 activation and thereby suppresses STAT3 signaling.	Indirect Inhibition	(126)	Preclinical studies	None
PZH2108	Directly targeting STAT3	Short-term treatment inhibits STAT3 phosphorylation, whereas prolonged exposure decreases total STAT3 protein levels.	Direct Inhibition	(54)	Phase II	None identified
Platycodon grandiflorus	Directly targeting STAT3	It reduces STAT3 phosphorylation and inhibits STAT3 activation in preclinical tumor models.	Direct Inhibition	(125)	Preclinical studies	None
Frankincense and Myrrh Extracts	SHP-1 activator	It induces SHP-1 and thereby indirectly inhibits STAT3 activation.	Indirect Inhibition	(102)	Preclinical studies	None
Piperlongumine	Directly targeting STAT3	It inactivates STAT3 by inhibiting its binding to the pY peptide ligand.	Direct Inhibition	(127)	Preclinical studies	None
Ganoderma lucidum Extract	Targeting upstream regulators	It inhibits the activation of STAT3 by suppressing the secretion of IL-6.	Indirect Inhibition	(32)	Preclinical studies	None
Cucurbitacin I	Targeting upstream regulators	It indirectly inhibits the STAT3 pathway by suppressing JAK2.	Indirect Inhibition	(25)	Preclinical studies	None
Resveratrol	Directly targeting STAT3	It suppresses STAT3 signaling, including reduced STAT3 acetylation/activation, in addition to broader antitumor regulatory effects.	Direct Inhibition	(25, 61)	Phase I/II	NCT00578396 (I) NCT00433576 (I) NCT00920803 (I) NCT02261844 (I/II)
Quercetin	Targeting upstream regulators	It inhibits STAT3 by suppressing the kinase activity of JAK2.	Indirect Inhibition	(15, 38, 61)	Phase I/II	NCT05680662 (I) NCT06615752 (I/II)
Cryptotanshinone	Directly targeting STAT3	It inhibits the phosphorylation of STAT3 by binding to its SH2 domain	Direct Inhibition	(15, 61, 128)	Preclinical studies	None
2-Methoxystypandrone	Targeting upstream regulators	It inhibits STAT3 by suppressing Janus kinase 2 and IkappaB kinase.	Indirect Inhibition	(15)	Preclinical studies	None
β-Elemene	Targeting upstream regulators	It inhibits STAT3 by suppressing the kinase activity of JAK2	Indirect Inhibition	(129)	Phase II	NCT03123484 (II)
Nobiletin	Targeting upstream regulators	It inhibits upstream targets (such as EGFR, JAK2) or regulates related molecules (such as miR-197), thereby reducing the level of phosphorylated STAT3.	Indirect Inhibition	(42)	Preclinical studies	None
Yifei Sanjie Formula	Regulating upstream metabolic processes	It reduces p-STAT3-associated signaling, at least in part by modulating bile-acid/metabolic pathways and related upstream regulators in lung cancer models.	Indirect Inhibition	(48)	Pilot randomized clinical study	None identified

Emerging biotechnology-based approaches provide additional opportunities to target STAT3 with greater mechanistic precision. Peptide- and peptidomimetic-based strategies, including phosphopeptides, SH2-binding peptides, aptamers, and cell-penetrating STAT3-targeting peptides, are designed to disrupt STAT3 dimerization, nuclear translocation, or transcriptional activity (25, 40, 61, 130, 131). These approaches are conceptually attractive because they directly target key structural or functional interfaces of STAT3, although delivery and pharmacokinetic limitations remain important barriers to translation (**Table 4**).

**Table 4:** Peptide- and peptidomimetic-based approaches targeting the STAT3 pathway.

Agent	Target level	Representative mechanism	Inhibition type	References	Clinical stage	NCT number
Phosphopeptide and peptidomimetic inhibitors	Directly targeting STAT3	Mimic the phosphotyrosine-containing STAT3-binding motif and competitively occupy the STAT3 SH2 domain, thereby disrupting dimerization, DNA binding, or nuclear translocation.	Direct Inhibition	(25)	Preclinical studies	None
PY*LKTK	Directly targeting STAT3	A phosphopeptide SH2-domain inhibitor that disrupts STAT3 phosphotyrosine-dependent interactions and inhibits STAT3 DNA binding and transcriptional activity.	Direct Inhibition	(61)	Preclinical studies	None
STAT3-binding aptide (APTSTAT3)	Directly targeting STAT3	Specifically binds STAT3; the cell-permeable derivative inhibits STAT3 phosphorylation and downregulates STAT3 target-gene expression.	Direct Inhibition	(130)	Preclinical studies	None
Cell-penetrating STAT3-targeting peptide	Directly targeting STAT3	A membrane/cell-penetrating peptide aptamer that interacts with a STAT3 dimerization-related interface, reduces STAT3 phosphorylation, and suppresses downstream STAT3 signaling.	Direct Inhibition	(130)	Preclinical studies	None

Nucleic acid-based approaches represent a second major biotechnology modality. These include antisense oligonucleotides, siRNAs, decoy DNA-binding sites, decoy oligodeoxynucleotides, danvatirsen (AZD9150), and miR-197-based strategies, all of which aim to reduce STAT3 expression or function at the RNA or transcriptional level (15, 120, 131). These strategies are attractive because they offer the possibility of highly specific pathway inhibition, although delivery, stability, and tissue penetration remain major translational challenges (**Table 5**).

**Table 5:** Nucleic acid-based strategies targeting the STAT3 pathway.

Agent	Target level	Representative mechanism	Inhibition type	References	Clinical stage	NCT number
Antisense oligonucleotides (ASOs)	Directly targeting STAT3	It inhibits the translation process of STAT3 mRNA by directly binding to it.	Direct Inhibition	(25)	Preclinical studies	None
siRNA	Directly targeting STAT3	It specifically inhibits the expression of STAT3 protein by down-regulating the mRNA level of STAT3.	Direct Inhibition	(25, 131)	Phase I	NCT04995536 (I)
STAT3 decoy oligonucleotides	Directly targeting STAT3	It directly binds to phosphorylated STAT3 (pSTAT3) and functions by competitively inhibiting the binding of endogenous STAT3 to the corresponding cis-elements (response elements) in the genomic DNA.	Direct Inhibition	(25, 130)	Phase 0	NCT00696176 (0)
Danvatirsen (AZD9150)	Directly targeting STAT3	It achieves this by binding to the specific sequence of STAT3 mRNA, thereby inducing its degradation and reducing the synthesis of STAT3 protein.	Direct Inhibition	(11, 15, 18, 40, 119)	Phase I/II	NCT01563302 (Ib) NCT02499328 (Ib/II) NCT02546661 (Ib) NCT03421353 (Ib/II) NCT02983578 (II) NCT05814666 (II)
miR-197 mimic	Targeting upstream factors	It suppresses STAT3 phosphorylation by targeting CKS1B expression	Indirect Inhibition	(10)	Preclinical studies	None

The strongest rationale for targeting STAT3 may ultimately lie not in monotherapy, but in rational combination treatment. Because STAT3 contributes to both tumor-intrinsic and immune-cell-intrinsic immune resistance, its inhibition may sensitize tumors to therapy at multiple levels (6, 7). By reducing PD-L1 expression, attenuating myeloid suppression, restoring antigen presentation, and weakening Treg- and TAM-dominant suppressive circuits, STAT3 blockade may enhance the efficacy of immune checkpoint inhibitors while also limiting the emergence of resistance. This provides a strong biological basis for combining STAT3-directed agents with immune checkpoint blockade, chemotherapy, or other targeted therapies. Clinically, this concept has already entered evaluation, as illustrated by studies of the STAT3 antisense oligonucleotide danvatirsen in combination with durvalumab in advanced solid tumors and recurrent/metastatic HNSCC (132), and by ongoing evaluation with pembrolizumab in recurrent/metastatic HNSCC (NCT05814666). Combination with chemotherapy is also supported by studies of ruxolitinib plus weekly paclitaxel in HER2-negative metastatic breast cancer (133). In parallel, STAT3 blockade is particularly attractive in targeted-therapy combinations because adaptive STAT3 activation can emerge as a bypass mechanism during kinase inhibition; reciprocal MEK-STAT3 signaling in pancreatic cancer provides one representative example and supports combined MEK plus STAT3 inhibition, which can remodel stromal inflammation and improve responsiveness to anti-PD-1 therapy in preclinical models (134, 135). Future progress in this area will likely depend on biomarker-guided strategies rather than empirical combination design. Candidate biomarkers may include baseline pSTAT3 or total STAT3 expression, IL-6/JAK/STAT3-related transcriptional signatures, and tumor contexts marked by myeloid-rich suppressive microenvironments, PD-L1/STAT3 co-activation, or adaptive STAT3 upregulation during targeted-therapy resistance (24, 136). Such an approach may better identify tumors in which STAT3 signaling is functionally dominant, and therefore more likely to benefit from combination regimens centered on STAT3 blockade.

## 7 Discussion and conclusions

STAT3 has emerged as a central regulatory hub in cancer immune escape. Its significance lies not only in its canonical role as a signal transducer and transcription factor, but also in its ability to connect tumor-cell-intrinsic survival programs with immune-cell dysfunction and metabolic niche formation. In tumor cells, STAT3 promotes stemness, anti-apoptotic signaling, checkpoint ligand expression, reduced antigenicity, and exosome-mediated immunoregulation. In immune cells, it drives Treg dominance, MDSC expansion, TAM polarization, and APC dysfunction. Through these combined actions, STAT3 converts inflammatory signaling into durable immune resistance.

This systems-level view also explains why STAT3 remains such an attractive therapeutic axis. Rather than controlling a single downstream phenotype, STAT3 organizes a broader immune-resistant tumor ecosystem. Accordingly, therapeutic targeting of STAT3 has the potential to simultaneously weaken tumor fitness, alleviate immunosuppression, and improve responsiveness to immunotherapy. The growing repertoire of small molecules, natural compounds, peptides, and nucleic acid-based agents underscores the translational momentum of this field (15, 25, 120).

At the same time, several major questions remain unresolved. First, the interaction of STAT3 with NF-κB, PI3K/AKT, MAPK, STAT1, and STAT5 is dynamic and spatially heterogeneous, yet most existing studies do not capture these relationships at sufficient resolution. Second, the activation state and functional consequences of STAT3 likely differ across tumor types and microenvironmental contexts, but large-scale clinically annotated datasets remain limited. Third, while the link between metabolism and STAT3 is increasingly recognized, more work is needed to define how STAT3 actively reprograms immune cell metabolism to drive exhaustion, myeloid suppression, and macrophage polarization. A related translational challenge is that systemic STAT3 inhibition may produce on-target toxicity because STAT3 is also required for normal immune, epithelial, and tissue homeostasis, as illustrated by the immunologic and multisystem abnormalities observed in human STAT3 deficiency syndromes (137, 138). In parallel, progress in the clinic will likely require more robust predictive biomarkers, including baseline pSTAT3 or total STAT3 levels, as well as IL-6/JAK/STAT3-related transcriptional signatures that may help identify tumors truly dependent on this axis (24, 139). Another emerging direction is the development of targeted protein degradation strategies, since recent PROTAC-based STAT3 degraders such as SD-36 have demonstrated highly selective STAT3 degradation and strong antitumor activity in preclinical models (140, 141). Finally, the clinical future of STAT3-targeted therapy will likely depend on biomarker-guided combinations rather than empiric monotherapy. Given the reciprocal coupling between STAT3 and tumor metabolism, combination strategies with metabolic inhibitors, including approaches targeting lactate/glycolysis, glutamine use, or fatty acid oxidation, may represent an especially important future direction, particularly in settings of CD8+ T-cell exhaustion and myeloid suppressive reprogramming (85, 142).

Future progress will depend less on viewing STAT3 as a standalone pathway than on exploiting it as a multicellular organizer of immune-resistant tumor ecosystems. This will require not only more precise identification of where, when, and in which cell types STAT3 is most therapeutically actionable, but also the development of biomarker-guided and toxicity-aware therapeutic strategies that can incorporate emerging modalities such as PROTACs and rational combinations with metabolic inhibitors. Defining where, when, and in which cell types STAT3 is most therapeutically actionable will be essential for designing rational intervention strategies that can overcome immune escape and extend the benefit of cancer immunotherapy.
